# Robust antibody responses in 70–80-year-olds 3 weeks after the first or second doses of Pfizer/BioNTech COVID-19 vaccine, United Kingdom, January to February 2021

**DOI:** 10.2807/1560-7917.ES.2021.26.12.2100329

**Published:** 2021-03-25

**Authors:** Sathyavani Subbarao, Lenesha A Warrener, Katja Hoschler, Keith R Perry, Justin Shute, Heather Whitaker, Michelle O’Brien, Frances Baawuah, Paul Moss, Helen Parry, Shamez N Ladhani, Mary E Ramsay, Kevin E Brown, Gayatri Amirthalingam

**Affiliations:** 1 Immunisation and Countermeasures Division, National Infection Service, Public Health England, London, United Kingdom; 2 Virus Reference Department, National Infection Service, Public Health England, London, United Kingdom; 3 Statistics, Modelling and Economics Department, National Infection Service, Public Health England, London, United Kingdom; 4 Brondesbury Medical Centre, Kilburn, London, United Kingdom; 5 Institute of Immunology and Immunotherapy, University of Birmingham, Edgbaston, United Kingdom; 6 Paediatric Infectious Diseases Research Group, St. George’s University of London, London, United Kingdom

**Keywords:** COVID-19, COVID-Vaccine, Antibody, Spike Protein, Immunity, Pfizer/BioNtech

## Abstract

Sera were collected from 185 adults aged ≥ 70 years in London to evaluate the immune response to COVID-19 vaccines. A single dose of Pfizer/BioNtech vaccine resulted in > 94% seropositivity after 3 weeks in naïve individuals using the Roche Spike antibody assay, while two doses produced very high spike antibody levels, significantly higher than convalescent sera from mild-to-moderate PCR-confirmed adult cases. Our findings support the United Kingdom’s approach of prioritising the first dose and delaying the second dose of COVID-19 vaccine.

In the United Kingdom (UK), the Joint Committee on Vaccination and Immunisation (JCVI) recommended extending the interval between coronavirus disease (COVID-19) vaccine doses from the authorised 3–4 weeks up to 12 weeks in order to maximise the roll-out of the first dose of vaccine to those at highest risk of death due to COVID-19 [1]. The COVID-19 vaccine responses after extended immunisation schedules (CONSENUS) evaluation aimed to assess immune responses to the extended immunisation schedule which was implemented across the UK from 8 December 2020. In this report, we present severe acute respiratory syndrome coronavirus 2 (SARS-CoV-2) antibody responses in the first 185 adults aged 70–90 years, recruited from the end of January 2021 through North London primary care networks, who were tested ca 3 weeks either after their first or second Pfizer/BioNTech (Mainz, Germany) vaccine dose received as part of the national programme. Responses were compared with 100 convalescent samples collected from clinically mild-to-moderate PCR-confirmed adult COVID-19 cases, ca 3–6 weeks after onset of symptoms.

## Serological testing

Serum samples were tested with five different antibody assays: two for antibodies against the nucleoprotein (N) (SARS-CoV-2 IgG assay, Abbott, Illinois, United States and Elecsys Anti-SARS-CoV-2 total antibody assay, Roche Diagnostics, Basel, Switzerland [2,3]) to identify prior SARS-CoV-2 infection, and three for antibodies against the spike (S) protein to assess vaccine response (Roche immunoassay, Elecsys Anti-SARS-CoV-2 S total antibody assay, Roche Diagnostics; an in-house receptor binding domain (RBD) indirect IgG ELISA [4]; and a lateral flow total antibody device (LFD), Fortress Diagnostics, Antrim, UK); the latter is currently used in the national Real-time Assessment of Community Transmission (REACT) study in the UK [5]. For the Abbott assay, results were expressed as a cut-off index (positive ≥ 1.4). Roche anti-N IgG results were expressed as a cut-off index (positive ≥ 1.0), and anti-S IgG as arbitrary units (au)/mL (positive ≥ 0.8 au/mL). For the RBD assay, results were expressed as an index calculated as the ratio test:negative (positive ≥ 5.0). For the LFD, 10 μL of serum were directly applied to the cartridge in the testing laboratory using the blood sample obtained through venepuncture. The devices were read by three independent observers and a consensus result derived. Only the IgG result was scored. All commercial assays were performed according to the manufacturer’s instructions.

## Vaccine responses

Seropositivity with the Roche anti-N-antibody assays was interpreted as evidence of previous infection, while lack of antibody to nucleoprotein and presence of spike protein antibody indicated vaccine response.

Fifteen of the 185 individuals (8%) were nucleoprotein antibody-positive using the Roche anti-N assay, including 10 who were also positive using the Abbott assay. Previous studies have shown that the sensitivity of the Abbott assay declines more rapidly compared with the Roche assay, indicating that those who were negative in the Abbott assay had been infected more than 3 months previously [6]. The nucleoprotein antibody seropositivity of 8% in our cohort is similar to community seroprevalence of 11% among ≥ 70-year-old blood donors between 18 January and 14 February 2021 in London [7].

**Figure 1:**
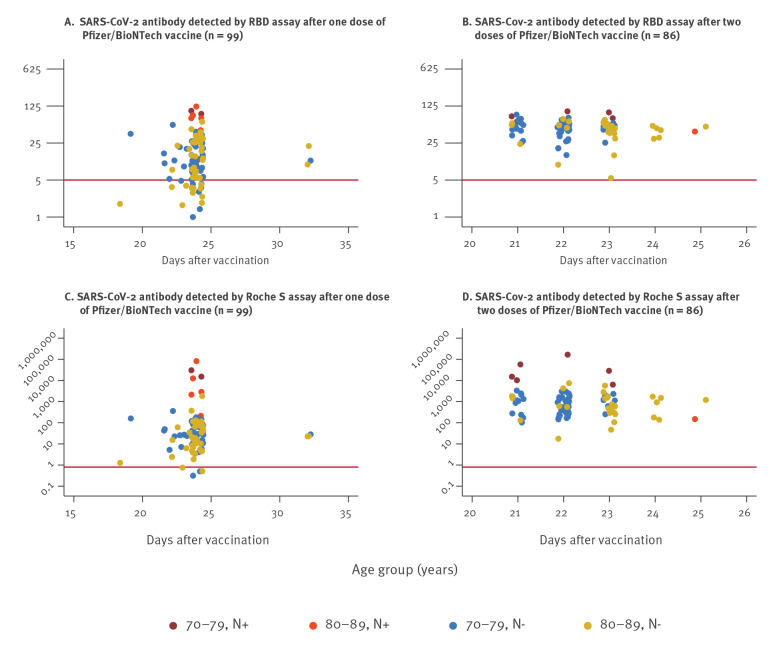
SARS-CoV-2 antibody levels in RBD and Roche S assays by age group, N antibody status and vaccine dose, London, United Kingdom, January–February 2021 (n = 185)

In this cohort, 99 individuals were enrolled after receiving one dose of Pfizer/BioNTech vaccine and 86 individuals were enrolled after receiving two doses 3 weeks apart. All individuals had a blood test ca 3 weeks after vaccination. In those who had received their first dose of vaccine (n = 99), sera were collected at day 0 and days 18–33 and in those who received two doses (n = 86), sera were only collected between days 21 and 25 after their second dose. All 86 individuals were spike protein antibody-positive in all three assays. After two vaccine doses, antibody titres were significantly higher in those with prior SARS-CoV-2 infection (nucleoprotein antibody-positive) compared with previously uninfected individuals (twofold by RBD; 20-fold by Roche S for those aged 70–79 years, Figure 1 and Table 1). There was no evidence of a boosting response to the second dose of vaccine in those who were previously infected with SARS-CoV-2.

N+: nucleoprotein-positive; N−: nucleoprotein-negative; RBD: receptor-binding domain assay; S: spike protein assay; SARS-CoV-2: severe acute respiratory syndrome coronavirus 2.

The horizontal line indicates the cut-off between positive and negative for each of the assays.

**Table 1:** RBD assay and Roche S measurements and Fortress lateral flow outcomes by age group, 3 weeks after first or second dose Pfizer/BioNTech vaccine, London, United Kingdom, January–February 2021 (n = 185)

Pfizer/BioNTech dose	Age group (years)	Total	RBD		Roche S		Lateral flow
			Number of positives	Geometric mean antibody concentrations (95% CI)	Number of positives	Geometric mean antibody concentrations (95% CI)	Number of positives
Roche N-negative							
1	70–79	49	42	11.9 (9.3–15.2)	47	27.1 (18.2–40.4)	40
1	80–89	43	29	10.1 (7.4–13.9)	40	19.6 (11.5–33.5)	31
2	70–79	50	50	45.2 (40.5–50.3)	49	740.4 (564.1–971.8)	50
2	80–89	28	28	39.7 (31.7–49.8)	28	640.3 (373.3–1,098.1)	28
Roche N-positive							
1	70–79	2	2	98.1 (61.8–155.9)	2	23,338 (746.8–729,398)	2
1	80–89	5	5	80.8 (46.5–140.2)	5	5,034.4 (252.4–100,431)	5
2	70–79	7	7	76.2 (63.9–90.8)	7	17,998 (4,378.7–73,982)	7
2	80–89	1	1	41.3	1	150	1
Convalescent sera							
Not applicable	15–98	100	91	29 (23.1–36.4)	93	31 (20.3–47.3)	Not done

CI: confidence interval; N: nucleoprotein; RBD: receptor-binding domain assay; S: spike protein assay.

Of the 92 individuals tested 3 weeks after their first dose of Pfizer/BioNTech vaccine and with no evidence of previous SARS-CoV-2 infection, 71 (77%) were positive in the RBD assay, 71 (77%) by LFD and 87 (95%) in the Roche anti-S assay. Antibody levels were slightly lower among ≥ 80-year-olds than 70–79-year-olds in both quantitative assays but the difference was not statistically significant (Table 1).

## Comparing responses to vaccine and natural infection

**Figure 2:**
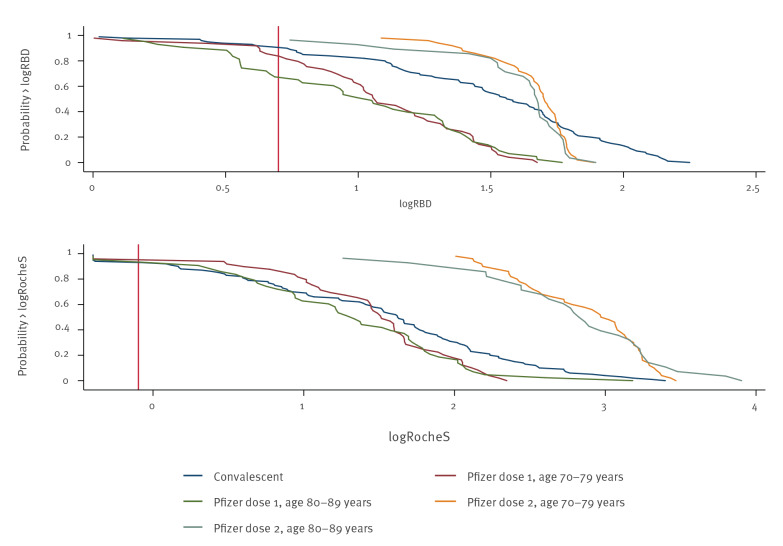
Reverse cumulative distribution plot of log (base 10) RBD or Roche S assay measurements in N-negative CONSENSUS participants by vaccine and convalescent sera, indicating relative antibody levels, London, United Kingdom, January–February 2021 (n = 285)

RBD: receptor-binding domain assay; S: spike protein assay.

Geometric mean antibody concentrations in those with and without prior infection were compared with 100 convalescent sera taken 3–6 weeks after PCR-confirmed infection among 15–98-year-olds (mean age: 50 years). Antibody levels after one vaccine dose in previously uninfected individuals were lower than convalescent sera using the RBD assay but similar when using the Roche anti-S assay (Figure 2 and Table 2).

Vertical line: cut-off between positive and negative for each assay.

**Table 2:** Geometric mean ratio of RBD and Roche S assay responses, vaccine group (3 weeks post dose 1 or 2) compared with convalescent sera (3–6 weeks post onset), London, United Kingdom, January–February (n = 285)

Vaccine group	Geometric mean ratio of RBD responses (95% CI) Reference group: convalescent	Geometric mean ratio of Roche S responses (95% CI) Reference group: convalescent
Dose 1, N-negative	0.4 (0.3–0.5)	0.8 (0.5–1.2)
Dose 1, N-positive	2.9 (1.5–5.9)	251.6 (67.3–940.9)
Dose 2, N-negative	1.5 (1.1–1.9)	22.7 (13.6–37.7)
Dose 2, N-positive	2.4 (1.3–4.7)	319 (92.4–1,101.8)

CI: confidence interval; RBD: receptor-binding domain assay; S: spike protein.

After two vaccine doses, antibody concentrations in previously uninfected individuals were significantly higher in the vaccinated group than convalescent sera using either S-antibody assay, with > 10-fold higher concentrations in the Roche anti-S assay. In previously infected individuals, a single vaccine dose produced significantly higher antibody levels than convalescent sera. Two vaccine doses, however, did not significantly increase the geometric mean ratio (Table 2).

## Discussion

Unlike North American and many European countries that vaccinated according to the licensed schedule, the JCVI in the UK recommended prioritising the first dose of vaccine and delaying the second dose for up to 12 weeks. This decision was made at a time when there was a rapid increase in COVID-19 cases, hospitalisations and deaths in the UK [3] and a single dose of Pfizer/BioNtech COVID-19 vaccine was estimated to provide 90% protection against COVID-19 after 2 weeks [2]. The policy was supported by modelling suggesting vaccinating a greater number of people with a single dose would prevent more deaths and hospitalisations than vaccinating fewer people with two doses [4].

Our findings demonstrate that adults aged ≥ 70 years mount robust antibody responses 3 weeks after a single dose of the Pfizer/BioNtech vaccine, with more than 94% previously uninfected individuals seroconverting after 3 weeks using the Roche S assay. These results are particularly important given the limited data that are available for older adults in prelicensure COVID-19 vaccine trials.

Antibody responses after a single vaccine dose were significantly higher in previously infected vaccinees, consistent with similar reports in younger healthcare workers [8]. Two doses of vaccine produced very high spike antibody levels, with significantly higher antibody levels in those with prior SARS-CoV-2 infection. In previously infected individuals, however, there was no evidence of boosting to the second vaccine dose when administered 3 weeks following the first dose. This finding supports the approach adopted in some countries, such as France recommending a 3-week schedule, to offer only one dose of vaccine to those with previously confirmed SARS-CoV-2 infection [9]. When compared with convalescent sera from clinically mild-to-moderate PCR-confirmed cases in adults, antibody levels were significantly higher after two doses of vaccine in those aged ≥ 70 years and also after a single dose of vaccine in previously infected individuals.

## Conclusion

Our findings provide additional support for the UK approach of prioritising the first dose of vaccine and is consistent with recent real-world data demonstrating high protection after one dose against SARS-CoV-2 infection, COVID-19, hospitalisation and deaths in the older population in the UK [10] and Israel [11]. The data are also in keeping with community seroprevalence data in blood donors showing a rapid rise in prevalence of spike protein antibody-positive but nucleoprotein-antibody negative adults [12]. Antibody responses in our cohort were significantly higher compared with recent estimates using self-collected LFDs in the vaccinated people in the community, even when compared with LFD results in this analysis, suggesting that this collection method may not be suitable for assessing vaccine responses through home testing for older adults [13]. Further studies are ongoing to assess antibody and cellular responses as well as antibody waning in adults and older adults receiving extended-interval schedules with any COVID-19 vaccine.
